# Decoupling Dissolution and Biological Kinetics in Polycaprolactone‐Based Denitrification: Direct Determination of Yield and Maximum Specific Growth Rate

**DOI:** 10.1002/wer.70439

**Published:** 2026-06-10

**Authors:** Dorsa Barkhordari, Jithin Mathew, Basem Haroun, Lars Rehmann, Sudhir Murthy, Tanush Wadhawan, Domenico Santoro

**Affiliations:** ^1^ Department of Chemical and Biochemical Engineering, Thompson Engineering Building Western University London ON Canada; ^2^ Water Research Center Lambton College Sarnia ON Canada; ^3^ NEWhub Water Corporation Herndon Virginia USA; ^4^ Dynamita SAS Sigale France

**Keywords:** biodegradable polymers, biomass yield, denitrification, maximum specific growth rate, polycaprolactone, solid‐phase carbon source

## Abstract

Solid‐phase denitrification using biodegradable polymers such as polycaprolactone (PCL) is increasingly proposed as a sustainable alternative to conventional soluble carbon dosing. However, reported kinetic and stoichiometric parameters for PCL‐based systems are often confounded by mass‐transfer and dissolution limitations, hindering accurate determination of biological kinetics. In this study, the dissolution step was explicitly decoupled from microbial growth by presolubilizing PCL prior to use, enabling the direct experimental determination of biomass yield (Y) and maximum specific growth rate (μ_max_) under strictly anoxic batch conditions. Methanol was investigated in parallel as a benchmark soluble carbon source. Yield assays conducted over 12 independent replicates produced statistically similar yields of 0.35 ± 0.08 and 0.38 ± 0.08 g COD per g COD for methanol and PCL, respectively, indicating comparable stoichiometric efficiency for biomass synthesis. Dynamic nitrate depletion profiles were resolved through high‐frequency batch testing and fitted using SUMO process modeling. The resulting μ_max_ values were 1.3 day^−1^ for methanol and 2.1 day^−1^ for presolubilized PCL, indicating that when carbon availability is not limited by dissolution, under the tested decoupled batch conditions, PCL‐derived substrates can support higher growth rates than conventional methanol. Long‐term acclimation tests showed lower net biomass accumulation with PCL, attributable to its slower carbon release rather than to microbial capacity limits. These results provide the first direct determination of true biological kinetic parameters for PCL‐based denitrification, independent of polymer hydrolysis effects. The findings demonstrate that the apparent kinetic constraints previously reported for solid PCL systems primarily reflect physicochemical mass‐transfer limitations and not biological capacity. This work establishes design‐ready kinetic parameters for integrating biodegradable polymers into predictive denitrification models, supporting the rational implementation of biodegradable polymeric carbon sources in sustainable nitrogen removal processes.

## Summary

- Presolubilized PCL achieved a biomass yield comparable to methanol while supporting a higher maximum specific growth rate.
- Solid PCL performance is limited by dissolution and mass‐transfer limitations rather than biological utilization capacity.
- Enhancing PCL dissolution can improve carbon availability, accelerate denitrification kinetics, and support more predictable treatment performance.
- PCL offers a more sustainable alternative to methanol for external carbon supplementation in denitrification systems.

## Graphical abstract

The graphical abstract compares methanol and presolubilized PCL as external carbon sources in anoxic batch denitrification tests. Although both substrates showed comparable biomass yields, presolubilized PCL supported a higher maximum growth rate than methanol. These results indicate that, when dissolution limitations are eliminated, PCL‐derived soluble carbon can be readily utilized by denitrifying bacteria and can provide reliable kinetic performance for evaluating biological denitrification.

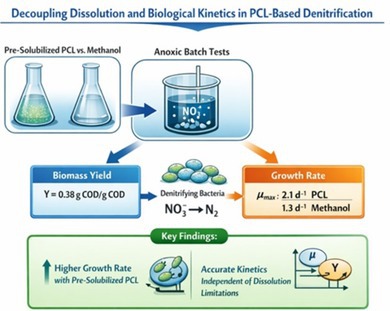

## 1 Introduction

In recent years, nitrate levels in water sources have increased mainly due to the use of nitrogen fertilizers and animal manure in agriculture. The discharge of excess nitrogen (as organic nitrogen, ammonia, nitrate, and nitrite) into water bodies negatively affects human health and aquatic life. When nitrate is ingested under conditions promoting nitroso compound formation, it can contribute to certain cancers and birth defects (Ward et al. 2018). In addition to methemoglobinemia, other diseases such as colorectal cancer, thyroid disease, and neural tube defects have been associated with nitrate in drinking water (Ward et al. 2018). Given these serious health concerns, the Environmental Protection Agency (EPA) has established regulatory limits of 10 mg/L for nitrate (NO_3_–N) and 1 mg/L for nitrite (NO_2_–N) in drinking water (United States Environmental Protection Agency (EPA) 2009). In addition to health risks, high nitrate levels in surface water can lead to eutrophication, harmful algal blooms, hypoxia, and fish deaths, all of which negatively affect water quality and ecosystem health (Wang et al. 2023). For these reasons, removing nitrogen compounds from wastewater is essential to protect both human and environmental health.

Various treatment methods (including physical, chemical, and biological processes) have been developed to remove nitrogen compounds from water. Among these, wastewater treatment plants prefer biological treatment processes due to their cost‐effectiveness in terms of energy consumption and chemical usage. For example, biological nutrient removal (BNR) has become the favored approach for nutrient removal. These systems modify treatment processes to help microorganisms convert nitrate into inert nitrogen gas (United States Environmental Protection Agency (EPA) 2009). Denitrification takes place in anoxic conditions; hence, it is influenced by several factors, including dissolved oxygen (DO), the type and availability of organic carbon, the COD/N ratio, temperature, and pH (Yang et al. 2012). The availability of carbon sources compared to nitrate/nitrite is often expressed as the COD or biochemical oxygen demand (BOD) to N ratio (COD/N, BOD/N). The COD/N ratio should be at least four for effective nitrate removal (Tchobanoglous 2014).

Nitrate reduction can be classified into two primary types based on the type of electron donors involved in the denitrification process: heterotrophic and autotrophic denitrification (Pang and Wang 2021). In autotrophic denitrification (ad), denitrifying bacteria utilize inorganic substances such as sulfide, sulfur, and hydrogen, as well as bicarbonates, as sources of carbon to reduce nitrate. These bacteria grow at a slower rate, making nitrate removal in this process slower than in heterotrophic denitrification, where bacteria grow faster. This method is suitable for wastewater with low organic carbon levels, but it needs a longer start‐up time and may lead to sulfate buildup in the system. The autotrophic denitrification can be divided into three main categories: hydrogen‐driven denitrification processes, anaerobic ammonium oxidation process (ANAMMOX), and sulfur‐based denitrification solid‐phase denitrification (SPD) process (Barkhordari et al. 2025).

Heterotrophic denitrification (HD) involves heterotrophic denitrifying bacteria, such as *Paracoccus pantotropha*, that rely on organic carbon sources, including methanol, acetate, or ethanol, as electron donors to convert nitrate, the electron acceptor, into nitrite and ultimately into nitrogen gas (Zhu et al. 2024). External carbon is often added into biological processes to support denitrification and remove nitrogen by supplying energy and carbon to heterotrophic bacteria, particularly when internal carbon sources are insufficient or depleted. Methanol is the most commonly used external carbon source because it is the most affordable in the United States (Mokhayeri et al. 2008). Research has shown that methanol used for denitrification is primarily consumed by specific heterotrophic bacteria, such as *Hyphomicrobium* spp. Consequently, understanding the kinetic parameters and stoichiometric characteristics of these microorganisms is essential for effectively designing and optimizing nitrogen removal systems that employ methanol addition (Dold et al. 2008). However, despite extensive research and widespread application of HD, there is still a limited understanding of how critical operational factors (such as the type and amount of carbon source) influence the efficiency of the process (Yang et al. 2012). Given the central role of carbon in supporting microbial activity, selecting an appropriate carbon source is critical for effective nitrate removal. Moreover, conventional carbon sources, such as methanol and ethanol, pose risks during handling and storage, as well as chemical dosing under highly variable flows and influent water qualities.

SPD is an emerging method for controlling nutrients in wastewater that has gained popularity due to the possibility of using biodegradable plastics in view of a more sustainable and circular approach to wastewater treatment. Indeed, by using biodegradable materials as the carbon source and biofilm carrier, SPD enables denitrifying microorganisms to gradually access and utilize the carbon through enzymatic hydrolysis. Controlled carbon release minimizes the possibility of overdosing or underdosing and makes process monitoring easier. Previous studies, after evaluating various synthetic biodegradable polymers and natural substrates, have concluded that polycaprolactone (PCL) is the one providing optimal performance (Wang and Chu 2016).

Polycaprolactone has been widely investigated among biodegradable polymers due to its balanced hydrolysis rate, mechanical stability, and ability to sustain long‐term denitrification performance across multiple reactor configurations (Li et al. 2016; Wu et al. 2013; Chu and Wang 2013; He et al. 2021); Jiang et al. 2020. Comparative studies have shown that PCL supports stable biofilm formation and high nitrate removal efficiencies while avoiding rapid depletion or instability observed with alternative polymers (Pan et al. 2013; Li et al. 2016; Chu and Wang 2013). The dissolution behavior of PCL is diffusion‐controlled and strongly influenced by temperature and surface area, resulting in gradual soluble COD release rather than immediate substrate availability (Pan et al. 2013; Zhang et al. 2016). Previous investigations have demonstrated that denitrification performance in solid PCL systems is frequently governed by polymer hydrolysis and mass‐transfer limitations rather than intrinsic microbial kinetics (Zhang et al. 2016; Clesceri et al. 1998). Consequently, isolating biological growth from dissolution effects is necessary to accurately quantify true kinetic parameters. This limitation motivated the decoupling strategy adopted in the present study, where PCL was presolubilized prior to batch testing to distinguish physicochemical carbon release from microbial metabolism.

Specifically, it has been reported that PCL behaves as a slow‐release carbon source, with soluble COD becoming available only after a dissolution step that proceeds through a diffusion‐controlled mechanism. Factors such as temperature and bead surface area significantly influence the release rate (Wang and Chu 2016). Because microorganisms can only utilize the dissolved fraction, carbon availability (rather than biological uptake) often governs the overall denitrification rate when PCL is supplied in solid form. Therefore, to properly characterize the biological kinetics of PCL, it is necessary to distinguish between limitations arising from dissolution and those associated with microbial growth. Denitrification kinetics (yield, half‐saturation, and growth rate) are crucial model inputs, as they define the denitrification rate in Monod‐based models. Having a thorough understanding of these parameters is essential, as they can vary widely depending on operational conditions, such as temperature, initial concentration of nitrate or nitrite, the type and concentration of the carbon source, and the diversity of the biomass. Therefore, efficient denitrification requires careful optimization of these parameters. Although many studies have reported yield calculations for carbon sources such as methanol, ethanol, and acetate, data on yield coefficients for denitrification with pure PCL are scarce. Reported biomass yield coefficients vary depending on the external carbon source. For methanol, yields are generally among the lowest, ranging from 0.21 to 0.45‐mg VSS/mg COD (Sun et al. 2009; Mahmoud et al. 2022; Peng et al. 2007). In comparison, ethanol yields are typically higher (0.42–0.53‐mg VSS/mg COD) (Mahmoud et al. 2022; Peng et al. 2007), whereas acetate yields show a broader but overall higher range (0.35–0.66‐mg VSS/mg COD) (Mokhayeri et al. 2008; Mahmoud et al. 2022; Peng et al. 2007; Mokhayeri et al. 2009; Dhamole et al. 2015). A yield coefficient of 0.21‐mg VSS/mg COD has been reported for PCL (Li et al. 2016). This yield, reported by Li et al. (2016) (0.44‐kg VSS per kg PCL), was normalized to the COD equivalent of PCL (2.11‐g O_2_/g PCL), corresponding to about 0.21‐mg VSS per mg COD. These differences are important, as higher biomass yields may enhance microbial growth and nitrate removal rates, but also increase sludge production and associated handling and disposal costs. Solid‐phase materials like PCL reportedly produce much lower biomass yields (≈0.21‐mg VSS/mg COD when normalized to COD) compared with soluble substrates. This has been attributed to the fact that, unlike soluble substrates such as methanol, PCL must dissolve before its carbon becomes available to microorganisms (Dhamole et al. 2015). However, most studies use PCL in solid form without prior solubilization. Because dissolution is both temperature‐dependent and relatively slow, the SCOD released from solid PCL tends to lack the immediate availability seen with soluble substrates. This means that many of the yield and kinetic values reported in the literature for PCL are apparent values, shaped more by limitations in carbon release than by the true biological behavior of the microbial community. Although soluble carbon sources like methanol, ethanol, and acetate have well‐established kinetics, far less is known about polymers such as PCL. The limited kinetic information available for PCL is difficult to compare across studies because most prior investigations focused on reactor‐level performance rather than isolating biological kinetics from solid‐phase carbon‐release effects (Table 1). As a result, the reported kinetics often combine dissolution and biological uptake effects, making it difficult to model PCL directly in denitrification models.

**TABLE 1:** Summary of the key denitrification studies that have used PCL, highlighting reactor configurations and corresponding performance outcomes.

Reactor type	Performance metrics	References
Continuous‐flow reactor	70% total *N* removal after 10 weeks	(Honda and Osawa 2002)
Packed‐bed bioreactor (fixed bed)	93% average nitrate removal efficiency at stable operation	(Chu and Wang 2013)
Fixed‐bed bioreactor (PCL‐packed column)	> 95% total *N* removal achieved (effluent NO_3_^−^–*N* < 3.7 mg/L; negligible NO_2_^−^/NH_4_^+^)	(Li et al. 2016)
Integrated solid‐phase denitrifying biofilter	Nitrate and suspended solids effectively removed (max. denitrification rate 3.80 g N/L·day; ~1.23 g N/L·day even at ≈8°C)	(Li et al. 2016)
Upflow fixed‐bed reactors (30%, 60%, 90% PCL fill)	> 98% NO_3_^−^–N removal for all packing ratios; minimal nitrite or ammonium accumulation	(He et al. 2021)
Vertical baffled solid‐phase reactor	Achieved denitrification rate ≈0.33 g N/L·day treating low C/N effluent; lower temperature reduced nitrate removal efficiency	(Jiang et al. 2020)

In this study, key biokinetic parameters for PCL‐driven denitrification were determined using presolubilized PCL to minimize the influence of solid‐phase carbon‐release limitations. This approach enabled a clearer comparison between PCL‐derived substrates and methanol under controlled anoxic batch conditions. The results are intended to support the incorporation of more representative PCL‐related kinetic parameters into denitrification models and to improve interpretation of polymer‐based denitrification systems in future laboratory and engineering studies. The present work also contributes to broader efforts toward sustainable wastewater treatment and resource‐efficient nitrogen removal. In particular, the use of biodegradable polymer‐based carbon sources aligns with the objectives of reducing reliance on hazardous liquid chemicals, improving process stability, and enabling more controlled carbon delivery in biological treatment systems. These aspects are consistent with the goals of improving water quality, reducing pollution, and enhancing the sustainability of water treatment infrastructure. Although the primary focus of this study is on the mechanistic determination of biokinetic parameters, the findings support the development of more efficient and predictable denitrification processes, which are relevant to global initiatives aimed at sustainable water management.

## 2 Materials and Methods

### 2.1 Laboratory Experiments

Polycaprolactone (PCL) beads used in this study were purchased as Polly Plastics Moldable Plastic Pellets from a commercial consumer supplier (Polly Plastics, sold via Amazon Canada). The material was used as received. Manufacturer specifications such as molecular weight, purity, and bead size distribution were not reported by the supplier and were therefore unavailable for inclusion in this study. Because the objective of the work was to evaluate experimentally generated soluble COD and the associated biological response, the conclusions were based on measured carbon release and denitrification behavior rather than on supplier‐reported polymer‐grade specifications.

Samples were analyzed for total suspended solids (TSS), volatile suspended solids (VSS), total COD (TCOD), soluble COD (SCOD), nitrate (NO_3_^−^), and nitrite (NO_2_^−^). Samples were filtered using 0.45‐μm membrane filters (VWR) for soluble components and 1.2‐μm filters for TSS and VSS analyses following Standard Methods (Clesceri et al. 1998). HACH methods were used to measure COD (Method 8000), nitrate (10020), and nitrite (Method 8153). pH was measured using a VWR portable pH meter. All samples were analyzed in duplicate to ensure accuracy. Blanks and standards were used to verify the accuracy of the results, and if standard results deviated by more than ±10% from expected values, the analysis was repeated. The summary of influent water quality parameters is presented in Table 2.

**TABLE 2:** Secondary effluent wastewater (from Middlesex Center WWTP in London, Ontario) characteristics (average ± standard deviation of nine samples).

Parameter	Concentration (mg/L)
Total COD (TCOD)	24.0 ± 3.5
Soluble COD (SCOD)	11.9 ± 1.2
NO_2_–N	0.0 ± 0.0
NO_3_–N	32.2 ± 1.6

Prior to the denitrification experiments, inoculum was obtained from the return activated sludge (RAS) stream of the Greenway Wastewater Treatment Plant in London, Ontario, and used for acclimation to the carbon sources. The inoculum was initially at 50‐mg/L VSS and was acclimated to the carbon source via successive batch cultivations. Specifically, the acclimation occurred in a lab‐scale nitrogen‐flushed sequencing batch reactor (SBR) system consisting of two gas‐washing bottles, each with a working volume of 5 L. The SBR operated on a 24‐h cycle with four stages: feeding (10 min), an anoxic reaction (23 h, maintained by mechanical stirring at 180 rpm), settling (40 min), and decanting (10 min). To ensure anoxic conditions, nitrogen gas was used to purge the bottles for 10 min. The sludge acclimation period lasted 21 days, during which the denitrification performance of the methanol and PCL systems was evaluated.

The secondary effluent wastewater (SEWW) used in the experiment contained 600‐mg/L soluble COD; methanol or predissolved PCL served as the carbon source in each reactor. In the system containing PCL, soluble PCL was first obtained by dissolving 200 g of PCL beads in 2 L of filtered secondary effluent for 7 days at 40°C. The soluble PCL stock was measured for COD, and the volume needed to reach 600‐mg/L COD was added to the reactor. Simultaneously, the effluent undergoing denitrification was further spiked with an appropriate concentration of acclimated inoculum (depending on the biological tests performed, see next sections) and supplemented with potassium nitrate (KNO_3_) to 100‐mg/L NO_3_–N, potassium dihydrogen phosphate (KH_2_PO_4_) to 10 mg/L, and ammonium chloride (NH_4_Cl) to 10 mg/L. Soluble COD, NO_3_–N, and NO_2_–N were measured regularly, with weekly VSS and TSS measurements to assess each carbon source's performance.

### 2.2 Maximum Specific Growth Rate Test

**FIGURE 1:**
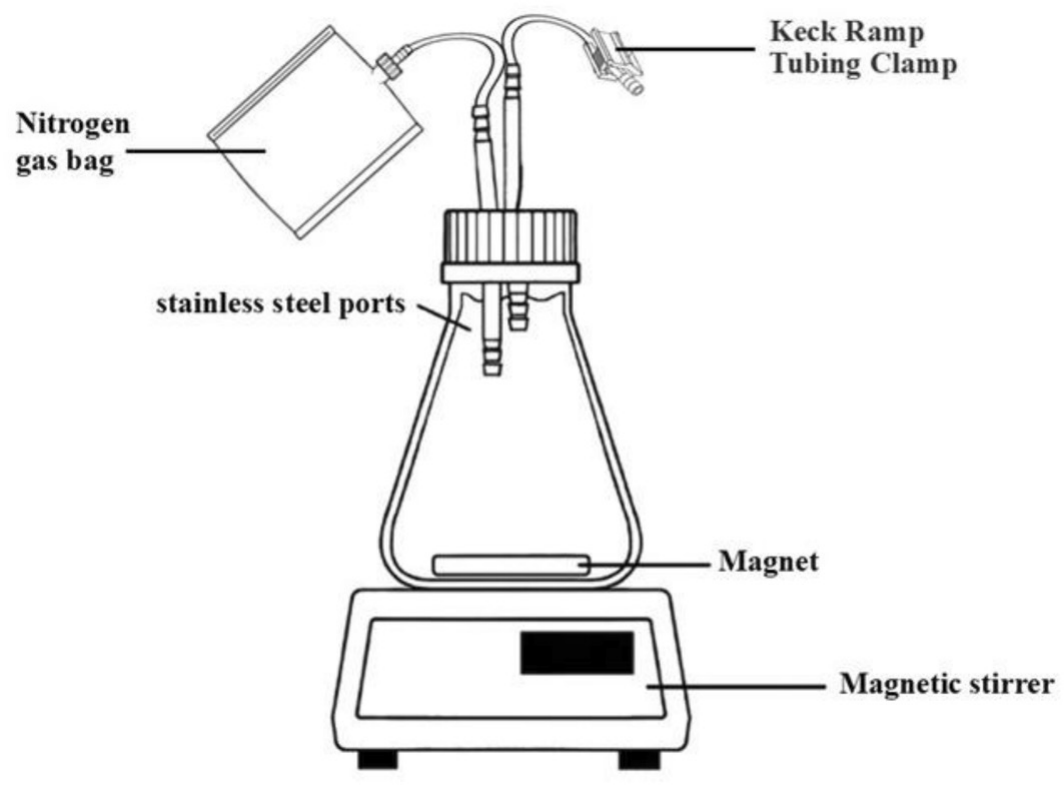
Schematic of the μ_max_ test setup using DURAN GL45 stainless steel multiport caps with N_2_ gas bag connection, sampling line (Keck Ramp Tubing Clamp), and magnetic stirring.

The maximum specific growth rate (μ_max_) tests were conducted in 1 L Erlenmeyer flasks, fitted with GL45 stainless steel 2‐port connection caps (DURAN Multiport Connector, Germany). One port was connected to an N_2_ gas bag to maintain anoxic conditions, whereas the second port served as a liquid sampling line. The sampling tubing was secured with a Keck Ramp Tubing Clamp, which served as a gas‐tight closure during operation and was opened only during syringe sampling (see Figure 1).

Two batch reactor systems were established: one with presolubilized PCL and one with methanol as the carbon source. For the PCL system, a stock solution was prepared by solubilizing 200 g of PCL beads in 2 L of FSEWW at 40°C for 48 h, and this solution was used as the carbon source.

It should be noted that in this study, the term “presolubilized PCL” refers operationally to a COD‐containing liquid stock prepared before biological testing and used in place of intact PCL beads. The purpose was not to claim complete molecular dissolution of the original polymer, but rather to provide the biomass with an already soluble carbon source and thereby decouple microbial growth from the rate‐limiting carbon‐release step associated with the solid polymer phase.

At time zero, substrate (methanol or PCL) was added to an initial concentration of approximately 600‐mg COD/L. RAS from Greenway WWTP (London, Ontario) was added to each system to provide the inoculum. Specifically, 12.2 mL of RAS was added to each reactor, yielding 70‐mg VSS/L initial biomass in each. Potassium nitrate (KNO_3_) was added to each system to provide an initial concentration of approximately 100‐mg NO_3_–N/L. Additionally, 10 mL of NH_4_Cl solution and 10 mL of KH_2_PO_4_ were added to each reactor as nutrient sources to prevent nutrient limitation. Nitrogen gas was bubbled through the liquid to strip dissolved oxygen. The final volume in each bottle was adjusted to 1 L with FSEWW sourced from the Middlesex Center WWTP in London, Ontario.

To ensure anoxic conditions, each bottle was purged with N_2_ gas for 10 min to remove dissolved oxygen. The bottles were then sealed with gas‐tight multiport caps, connected to nitrogen‐filled gas bags, and placed on magnetic stirrers to initiate mixing. Immediately after mixing began, the first sample was withdrawn to represent time zero. The batch tests were operated until complete nitrate depletion, which required 99 h for the PCL system and 138 h for the methanol system. During this period, 39 samples were collected from the PCL system and 54 from the methanol system at 2–4‐h intervals. The tests were performed at room temperature, consistent with conditions reported by Dold et al. (2008). Samples were taken using a syringe, then immediately filtered (using 0.45‐μm syringe filters) and analyzed for soluble COD, nitrite, and nitrate concentrations using HACH test kits and a HACH DR3900 Benchtop VIS Spectrophotometer. Total COD was analyzed simultaneously on unfiltered samples. VSS analysis was performed in triplicate, and the results were averaged. All measurements were made in duplicate and averaged.

Batch experiments were conducted to quantify microbial kinetics using PCL and methanol as carbon sources under controlled anoxic (denitrifying) conditions. To aid in interpreting the experimental data, dynamic simulations were performed using SUMO version 24.0.1 (Dynamita Company SAS, France) and the Sumo2 whole‐plant biokinetic model. Sumo2 is an advanced two‐step nitrification and denitrification framework derived with ammonia‐oxidizing bacteria (XAOB), nitrite‐oxidizing bacteria (XNOB), ordinary heterotrophic organisms (XOHO), and anoxic methanol utilizers (XMEOL). The model clearly separates nitrification into ammonia oxidation by XAOB and nitrite oxidation by XNOB. It also incorporates an extended denitrification pathway, enabling nitrite‐based denitrification under anoxic conditions. Model evaluation focused on the behavior of the anoxic methanol utilizers, the group of microorganisms responsible for methanol‐driven denitrification. These organisms grow exclusively under anoxic conditions, using nitrate or nitrite as terminal electron acceptors. The Sumo2 defines distinct kinetic and stoichiometric parameters for this group, including the maximum specific anoxic growth rate, half‐saturation constants, and aerobic/anoxic decay rates.

Since the Sumo2 model does not include PCL as an intrinsic substrate, methanol was used as a surrogate carbon source in simulations of both PCL and methanol batch assays. Each simulation was configured as a batch reactor with a working volume of 1 m^3^, consistent with the experimental setup. Aeration was disabled throughout each run to maintain strictly anoxic conditions. The initial concentrations of essential nutrients—including ammonia, nitrate, phosphorus, and the respective carbon source were set to match those measured at the start of the laboratory batch tests. For each scenario, the concentrations of nitrate and soluble COD (SCOD) were input using values directly obtained from the experimental data. Simulations were run for 7 days, with outputs recorded at 0.5‐h intervals.

The goal of the simulations was to reproduce the temporal profiles of nitrate, nitrite, ammonia, and carbon consumption observed in the laboratory batch tests and to estimate key biokinetic parameters governing methanol utilizers. Two specific parameters were targeted for calibration: (i) the initial concentration of methanol utilizers present at the beginning of each test, and (ii) the maximum specific anoxic growth rate of this biomass. Additional parameters, such as the substrate yield determined from an independent yield test and the decay rate, were held constant. Calibration was performed through the iterative adjustment of the target parameters until the modeled concentration profiles aligned with the experimental measurements.

### 2.3 Yield (Y) test

The procedure began with the preparation of two 5‐L gas‐washing bottles. Each reactor was filled with 5 L of SEWW obtained from the Middlesex center wastewater treatment plant located in London, Ontario, ensuring that the initial SCOD concentration was approximately 600 mg/L, and the nitrate concentration was around 100 mg/L. RAS from the Greenway WWTP (London, Ontario) was introduced into both bottles to achieve a VSS concentration of approximately 50 mg/L. Additionally, 50 mL of phosphate and ammonium chloride solutions were added as nutrient sources, and the pH was then adjusted to 7.0. Nitrogen gas was purged into the reactors to maintain anoxic conditions, preventing exposure to air. Once the initial reactor mixture was prepared, the bottles were placed on magnetic stirrers for 24 h. After this mixing period, samples were collected to measure SCOD and nitrate concentrations in each bottle. The stirrers were then stopped, and the reactors were allowed to settle for 1 h. One liter of supernatant was carefully removed from each reactor, and the volume removed was replaced with a fresh stock solution and SEWW to restore the original concentrations of 600‐mg/L SCOD and 100‐mg/L nitrate, bringing the total volume in each bottle back to 5 L. This procedure was repeated daily for 3 weeks. After the end of the third week of acclimation, a yield test was conducted by measuring SCOD and nitrate concentrations hourly for 8 h under anoxic conditions. To prevent nitrogen intrusion during sampling, the nitrogen gas tank was briefly connected to the gas‐washing bottle via a flexible tube. This connection occurred only during sampling. A gentle flow of nitrogen was introduced into the bottle, creating slight pressure that displaced the liquid and allowed it to be withdrawn through the outlet without exposure to ambient air.

The test was used to determine the C/N ratio for methanol and PCL, defined as the quantity of methanol or PCL consumed per unit of nitrate removed. This ratio can subsequently be applied to calculate the yield coefficient of the methanol‐ or PCL‐utilizing heterotrophs (*Y*). To obtain this value, COD measurements must be taken from filtered samples (using 0.45‐μm syringe filters) collected from the batch reactor during the experiment. The C/N ratio for denitrification with methanol was determined by plotting SCOD against nitrate concentration, where the slope of the least squares regression line through the experimental data represents the amount of methanol or PCL required for nitrate removal (expressed as mg methanol per mg nitrate). The *Y* for the methanol‐ or PCL‐utilizing heterotrophs can be derived from the measured C/N ratio, based on the relationship between the COD consumed and the amount of nitrate reduced, as follows:

(1) $$\mathrm{COD}/N=\frac{2.86}{1-Y}\text{mgCOD}/\mathrm{mg}{\mathrm{NO}}_{3}-N$$

The constant 2.86 in Equation (1) represents the oxygen equivalent of nitrate‐nitrogen (g O_2_/g NO_3_–N) for complete denitrification to N_2_. In the present work, stoichiometric interpretation was performed on a COD‐equivalent basis. Because presolubilized PCL likely consisted of a mixture of biodegradable dissolved intermediates rather than a single analytically resolved compound, the yield analysis was based on measured COD/N relationships rather than on a balanced molecular reaction for one specific PCL‐derived substrate.

For detailed stoichiometric calculations of the electron requirements and COD/N ratios for each sequential denitrification step, including the derivation of intermediate COD equivalents, refer to the Supporting Information.

Rearranging Equation (1), we obtain Equation (2):

(2) $$Y=1-\frac{2.86}{\mathrm{COD}/N}\;\text{mgCOD}/\text{mgCOD}$$

## 3 Results and Discussion

**FIGURE 2:**
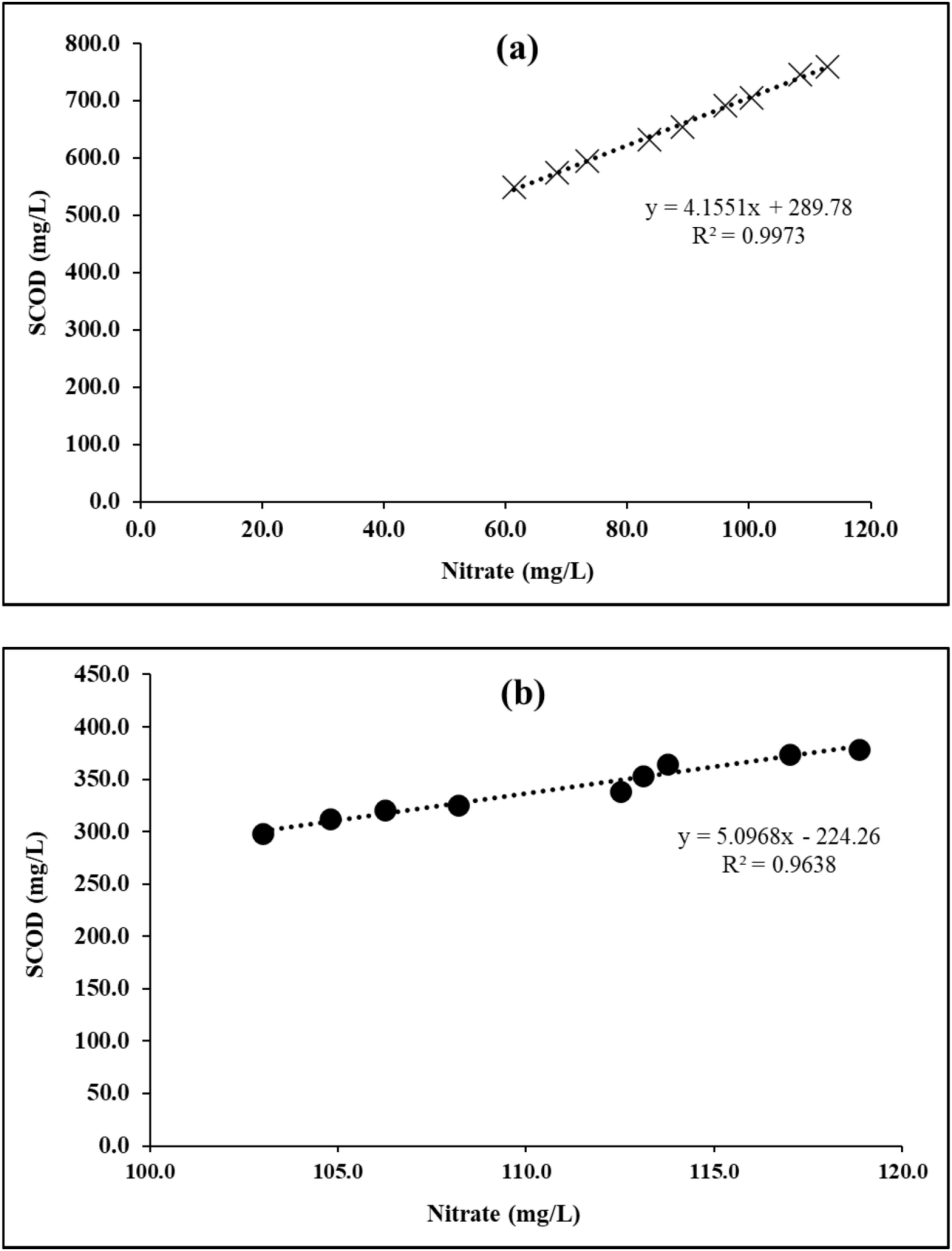
Regression of soluble COD against nitrate concentration for (a) methanol and (b) PCL.

Biological processes are governed by structured biokinetic relationships, typically represented through Monod‐type formulations, where growth rate depends on substrate availability, biomass concentration, and environmental conditions. In the present study, kinetic interpretation is therefore based on the estimation of key biokinetic parameters, specifically the maximum specific growth rate (μ_max_) and biomass yield (Y), rather than on empirical reaction‐order classification. Specifically, the stoichiometric yield of denitrifying biomass was estimated from COD/N relationships measured following sludge acclimation to methanol and PCL. Linear regression of soluble COD versus nitrate concentration produced COD/N slopes of 4.16 for methanol (*R* ^2^ = 0.997) and 5.10 for PCL (*R* ^2^ = 0.964) (Figure 2a,b). Nitrite accumulation was minimal during these tests, supporting the assumption that nitrate reduction proceeded predominantly toward complete denitrification in the liquid phase.

Substitution of these slopes into Equation (2) yielded biomass yield coefficients of *Y* = 0.31 g COD/g COD for methanol and *Y* = 0.44 g COD/g COD for PCL. To obtain statistically significant results, 12 additional yield tests were conducted to obtain COD/N ratios for each carbon source (results were averaged across replicates). The mean COD/N values were 4.41 ± 0.53 for methanol and 4.62 ± 0.56 for PCL, corresponding to yields of 0.35 ± 0.08 and 0.38 ± 0.08 g COD/g COD, respectively (Table 3).

**TABLE 3:** Average COD/N ratios and biomass yield coefficients for methanol and PCL, compared with the methanol benchmark reported by (Dold et al. 2008). (Mean values are reported as mean ± standard deviation based on 12 independent yield assays, and differences were assessed using a paired *t*‐test.)

Substrate	COD/N (mg COD/mg NO_3_–N)	Yield (g COD/g COD)	References
Methanol	4.74	0.40	(Dold et al. 2008)
	4.41 ± 0.53	0.35 ± 0.08	This study (averaged)
PCL	4.62 ± 0.56	0.38 ± 0.08	This study (averaged)

The average yield obtained for presolubilized PCL was comparable to the methanol benchmark (*Y* = 0.40 at 20°C) reported by Dold et al. (2008), indicating that once supplied as soluble COD, PCL‐derived substrate can support denitrifying biomass formation with similar stoichiometric efficiency to methanol. This observation is important because previously reported low PCL‐associated yields have often been obtained from systems using intact solid polymer, where substrate availability is constrained by upstream carbon‐release processes. The present results therefore suggest that low apparent yield values in solid PCL systems may partly reflect limited carbon availability rather than intrinsically poor biological conversion efficiency.

From a practical perspective, methanol yields faster denitrification kinetics, but PCL provides a stable, long‐term carbon release that supports ongoing biomass growth. This is especially advantageous in systems where slow‐release carbon is preferred, such as intermittent treatment systems in decentralized communities or agricultural runoff ponds.

Despite the comparable yield coefficients, long‐term acclimation data showed more biomass accumulation in the methanol system than in the PCL system. After 21 days, VSS increased from 60 to 410 mg/L in the methanol‐amended system, compared to an increase from 80 to 300 mg/L in the PCL system. Initially, this appears contradictory, since PCL's yield was comparable to methanol (0.38 vs. 0.40‐g COD/g COD (Dold et al. 2008)). However, biomass production depends on both yield and the maximum specific growth rate. Despite similar yields, the PCL‐amended system accumulated biomass more slowly. This suggests that PCL's carbon release is slower than methanol's (which is fully soluble and easily metabolized). Therefore, understanding the growth kinetics is crucial for interpreting these differences.

The use of the SUMO2 modeling framework allowed for a mechanistic representation of denitrification dynamics, consistent with established practices in wastewater process modeling. As such, the kinetic analysis presented here reflects intrinsic biological behavior under controlled conditions rather than simplified reaction‐order approximations. The higher μ_max_ obtained with presolubilized PCL compared with methanol likely reflects differences in metabolic pathways and energetic efficiency rather than carbon availability alone. Hydrolysis products derived from PCL consist primarily of low‐molecular‐weight fatty acids and oligomeric intermediates that can enter central metabolism through *β*‐oxidation and the tricarboxylic acid cycle, pathways broadly utilized by heterotrophic denitrifiers and associated with efficient ATP generation (Pang and Wang 2021; Wang and Chu 2016). In contrast, methanol utilization is typically restricted to specialized methylotrophic bacteria such as *Hyphomicrobium* spp., which require multistep oxidation pathways prior to assimilation, potentially limiting maximum growth rates (Dold et al. 2008). Although microbial community composition was not directly characterized in this study, previous work has shown that PCL‐fed systems enrich a more diverse heterotrophic consortium compared with methanol‐fed reactors (Chu and Wang 2013).

The maximum specific growth rate values obtained from denitrification experiments fitted with SUMO, with results summarized in Table 4. For methanol, the fitted μ_max_ was 1.3 day^−1^, nearly identical to the value reported by Dold et al. (2008). The PCL system exhibited a higher μ_max_ of 2.1 day^−1^. Despite the comparable yield coefficients, long‐term acclimation data showed more biomass accumulation in the methanol system than in the PCL system. After 21 days, VSS increased from 60 to 410 mg/L in the methanol‐amended system, compared to an increase from 80 to 300 mg/L in the PCL system. Initially, this appears contradictory since PCL's yield was comparable to methanol. However, biomass production depends on both yield and the maximum specific growth rate.

**TABLE 4:** Summary of maximum specific growth rate value obtained from SUMO simulation of this study, compared with literature values at 20°C (Dold et al. 2008).

Substrate	μ_max_ (day^−1^)	References
Methanol	1.3	This study
	1.3	(Dold et al. 2008)
PCL	2.1	This study

Although presolubilized PCL was used during controlled kinetic tests, differences in long‐term biomass accumulation may still arise from substrate‐specific metabolic allocation rather than dissolution limitations alone. Methanol represents a single‐carbon, fully soluble substrate that can be rapidly and consistently assimilated by methylotrophic denitrifiers, promoting stable biomass retention over extended acclimation periods (Dold et al. 2008). In contrast, PCL‐derived substrates comprise a mixture of fatty acids and oligomers that require additional intracellular processing via *β*‐oxidation prior to assimilation, potentially increasing maintenance energy demand and diverting a fraction of the consumed carbon toward respiration instead of biomass synthesis (Tchobanoglous 2014). Furthermore, systems utilizing polymer‐derived substrates may experience higher microbial turnover or endogenous decay due to fluctuations in substrate composition during acclimation, which can result in lower net VSS accumulation despite comparable apparent yield coefficients.

The Sumo2 model accurately replicates the observed denitrification behavior during batch testing, despite the need for a surrogate representation of PCL. The resulting calibrated parameters provided insights into the growth characteristics of methanol‐utilizing bacteria under these conditions and support the broader application of the model for evaluating carbon‐driven nitrogen removal processes.

From a practical perspective, biodegradable polymers such as PCL may be most relevant in applications where gradual carbon delivery is desirable, including polishing systems, decentralized treatment, or treatment scenarios subject to variable loading. However, several challenges remain for scale‐up, including polymer cost, long‐term release stability, possible accumulation of residual solids or fines, hydrodynamic nonuniformity in larger reactors, and the need for a better understanding of long‐term microbial adaptation. In addition, although biodegradable polymers may reduce reliance on conventional liquid carbon dosing, broader sustainability claims require life cycle, economic, and environmental evaluation beyond the scope of the present study.

The higher fitted μ_max_ obtained for presolubilized PCL does not by itself imply that PCL is universally superior to methanol as a denitrification carbon source. Methanol remains a well‐established, low‐cost soluble substrate with extensive full‐scale use. The relevance of PCL lies in a different operational niche, namely, its potential to provide controlled carbon release in solid‐phase applications where simplified dosing, reduced handling risks, or more gradual substrate delivery may be beneficial.

**FIGURE 3:**
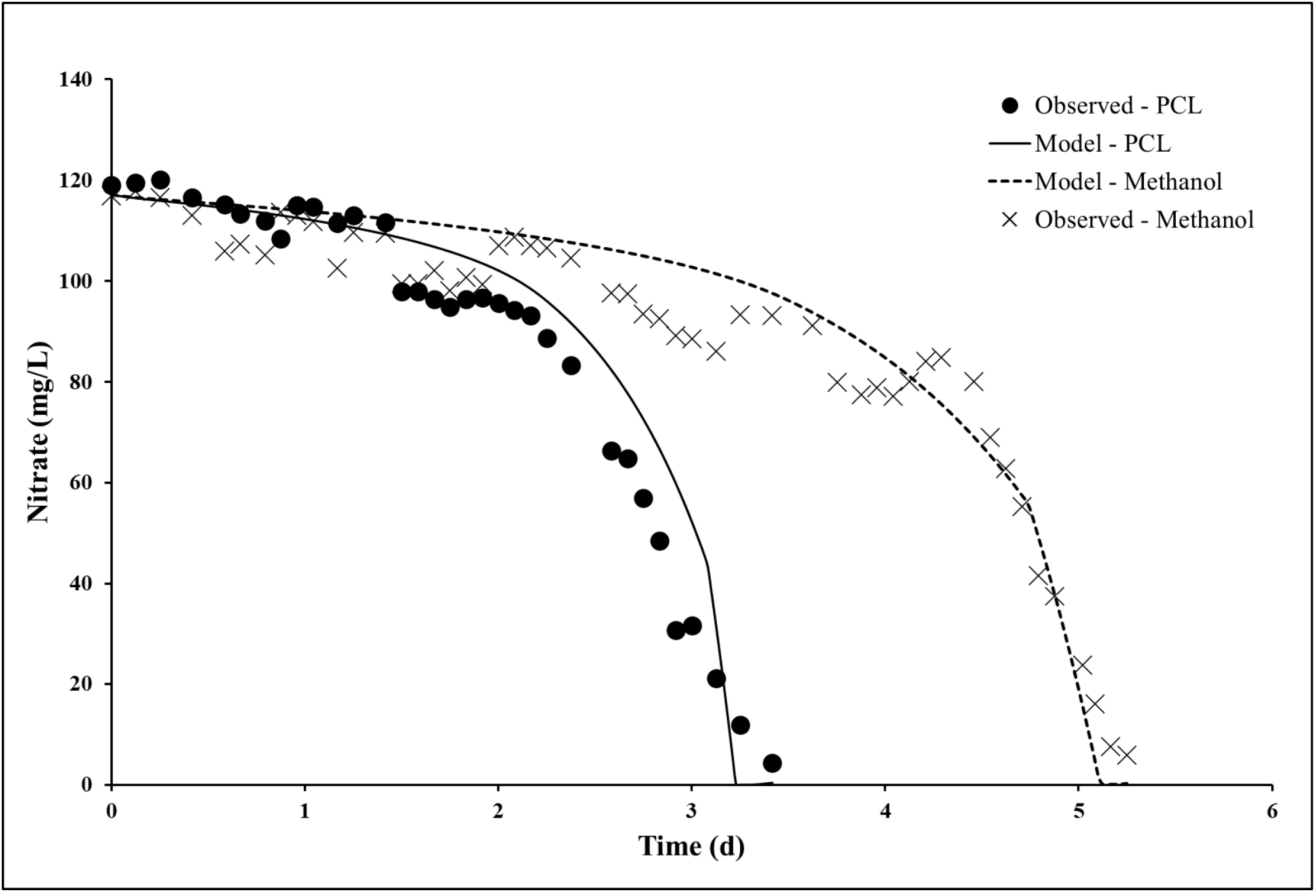
Experimental and modeled nitrate depletion profiles for methanol, and presolubilized PCL system.

It is important to note that, in this study, PCL was presolubilized; thus, microbial growth was not limited by polymer dissolution. Figure 3 shows the dynamic nitrate depletion profiles for the methanol and PCL systems, respectively, alongside the corresponding SUMO model simulations.

The nitrate depletion trend observed for methanol was qualitatively consistent with that reported by Dold et al. (2008). In contrast, the presolubilized PCL system exhibited a steeper nitrate depletion profile, corresponding to the higher fitted μ_max_ of 2.1 day^−1^ under the tested conditions. These results indicate that once solid‐phase carbon‐release limitations are minimized, PCL‐derived dissolved substrates can support rapid denitrifying growth.

## 4 Conclusions

A critical aspect of the present study is the use of presolubilized PCL to decouple biological kinetics from physicochemical limitations associated with solid‐phase carbon sources. In conventional systems using intact polymer media, the overall denitrification rate is often governed by upstream processes such as hydrolysis, dissolution, and mass transfer, which control the rate at which biodegradable carbon becomes available to microorganisms. By providing a presolubilized COD source, the present work effectively removes these limitations and enables direct observation of intrinsic microbial growth kinetics.

It is important to clarify that the term “presolubilized PCL” is used operationally to describe a COD‐containing liquid phase derived from polymer conditioning under elevated temperature and extended contact time. This approach does not imply complete molecular dissolution of the original polymer but rather ensures that carbon is available in a form that is immediately accessible to microbial metabolism. Under these conditions, mass‐transfer resistance between solid and liquid phases is minimized, and the measured μ_max_ values can be interpreted as representative of biological capacity rather than transport‐limited behavior.

The higher μ_max_ observed for PCL‐derived substrates compared with methanol suggests that, once dissolution constraints are removed, the nature of the carbon source plays an important role in determining microbial growth dynamics. PCL‐derived substrates are likely composed of a mixture of low‐molecular‐weight intermediates, including fatty acids and oligomers, which can enter central metabolic pathways such as *β*‐oxidation and the tricarboxylic acid cycle. In contrast, methanol utilization is typically restricted to specialized methylotrophic organisms and requires additional enzymatic steps prior to assimilation. These differences may contribute to the observed variation in growth rates. However, it should be emphasized that the present study does not include direct microbial community characterization, and therefore these interpretations remain mechanistic hypotheses consistent with established biochemical knowledge.

This study determined the biomass yield coefficient and maximum specific growth rate for PCL‐driven denitrification under conditions designed to minimize the influence of solid‐phase carbon‐release limitations. Average yield values of 0.35 ± 0.08 g COD/g COD for methanol and 0.38 ± 0.08 g COD/g COD for presolubilized PCL indicated comparable biomass conversion efficiency between the two substrates. The fitted μ_max_ values were 1.3 day^−1^ for methanol and 2.1 day^−1^ for presolubilized PCL under the tested anoxic batch conditions.

These results suggest that the relatively slow performance often reported for intact solid PCL systems is not necessarily caused by limited biological capacity but may instead reflect the influence of upstream carbon‐release and mass‐transfer constraints. Accordingly, interpretation of PCL‐based denitrification systems requires consideration of both microbial kinetics and the physicochemical processes that govern soluble carbon availability.

Although the results support the potential of biodegradable polymer‐derived substrates as alternatives to conventional soluble carbon sources in selected applications, broader assessment of their practical suitability will require further work on temperature effects, long‐term operation, microbial community dynamics, reactor‐scale implementation, environmental implications, and economic feasibility.

## Author Contributions

**Dorsa Barkhordari:** investigation, validation, formal analysis, writing – original draft. **Jithin Mathew:** investigation, writing – original draft, validation, methodology. **Basem Haroun:** investigation, writing – original draft, writing – review and editing, methodology. **Lars Rehmann:** conceptualization, funding acquisition, writing – review and editing, resources. **Sudhir Murthy:** conceptualization, funding acquisition, writing – review and editing, methodology, resources. **Tanush Wadhawan:** investigation, software, writing – review and editing. **Domenico Santoro:** conceptualization, investigation, funding acquisition, writing – original draft, writing – review and editing, methodology, validation, visualization, project administration, resources, formal analysis, supervision.

## Conflicts of Interest

The authors declare no conflicts of interest.

## Supporting information


**Table S1:** Stoichiometric COD/N ratios and electron requirements for sequential denitrification steps.

## Data Availability

The data that support the findings of this study are available from the corresponding author upon reasonable request.

## References

[wer70439-bib-0002] Barkhordari, D. , J. Mathew , B. Haroun , L. Rehmann , S. Murthy , and D. Santoro . 2025. “Wastewater Denitrification With Solid‐Phase Carbon: A Sustainable Alternative to Conventional Electron Donors.” Nitrogen 6, no. 2: 22. 10.3390/nitrogen6020022.

[wer70439-bib-0003] Chu, L. , and J. Wang . 2013. “Denitrification Performance and Biofilm Characteristics Using Biodegradable Polymers PCL as Carriers and Carbon Source.” Chemosphere 91, no. 9: 1310–1316. 10.1016/j.chemosphere.2013.02.064. 23545191

[wer70439-bib-0004] Clesceri, L. S. , L. S. Clesceri , American Public Health Association , American Water Works Association , and Water Pollution Control Federation , eds. 1998. Standard Methods: For the Examination of Water and Wastewater. 20th ed. American Public Health Association.

[wer70439-bib-0005] Dhamole, P. B. , S. F. D'Souza , and S. S. Lele . 2015. “A Review on Alternative Carbon Sources for Biological Treatment of Nitrate Waste.” Journal of The Institution of Engineers (India): Series E 96, no. 1: 63–73. 10.1007/s40034-014-0055-8.

[wer70439-bib-0006] Dold, P. , I. Takács , Y. Mokhayeri , et al. 2008. “Denitrification With Carbon Addition—Kinetic Considerations.” Water Environment Research 80, no. 5: 417–427. 10.2175/106143007X221085. 18605381

[wer70439-bib-0007] He, X. , S. Zhang , Y. Jiang , M. Li , J. Yuan , and G. Wang . 2021. “Influence Mechanism of Filling Ratio on Solid‐Phase Denitrification With Polycaprolactone as Biofilm Carrier.” Bioresource Technology 337: 125401. 10.1016/j.biortech.2021.125401. 34157434

[wer70439-bib-0008] Honda, Y. , and Z. Osawa . 2002. “Microbial Denitrification of Wastewater Using Biodegradable Polycaprolactone.” Polymer Degradation and Stability 76, no. 2: 321–327. 10.1016/S0141-3910(02)00028-9.

[wer70439-bib-0009] Jiang, L. , A. Wu , D. Fang , et al. 2020. “Denitrification Performance and Microbial Diversity Using Starch‐Polycaprolactone Blends as External Solid Carbon Source and Biofilm Carriers for Advanced Treatment.” Chemosphere 255: 126901. 10.1016/j.chemosphere.2020.126901. 32387904

[wer70439-bib-0010] Li, P. , J. Zuo , Y. Wang , J. Zhao , L. Tang , and Z. Li . 2016. “Tertiary Nitrogen Removal for Municipal Wastewater Using a Solid‐Phase Denitrifying Biofilter With Polycaprolactone as the Carbon Source and Filtration Medium.” Water Research 93: 74–83. 10.1016/j.watres.2016.02.009. 26897042

[wer70439-bib-0011] Mahmoud, A. , R. A. Hamza , and E. Elbeshbishy . 2022. “Enhancement of Denitrification Efficiency Using Municipal and Industrial Waste Fermentation Liquids as External Carbon Sources.” Science of the Total Environment 816: 151578. 10.1016/j.scitotenv.2021.151578. 34774960

[wer70439-bib-0012] Mokhayeri, Y. , R. Riffat , S. Murthy , W. Bailey , I. Takacs , and C. Bott . 2009. “Balancing Yield, Kinetics and Cost for Three External Carbon Sources Used for Suspended Growth Post‐Denitrification.” Water Science and Technology 60, no. 10: 2485–2491. 10.2166/wst.2009.623. 19923753

[wer70439-bib-0013] Mokhayeri, Y. , R. Riffat , I. Takacs , et al. 2008. “Characterizing Denitrification Kinetics at Cold Temperature Using Various Carbon Sources in Lab‐Scale Sequencing Batch Reactors.” Water Science and Technology 58, no. 1: 233–238. 10.2166/wst.2008.670. 18653959

[wer70439-bib-0014] Pan, Y. , B.‐J. Ni , and Z. Yuan . 2013. “Modeling Electron Competition Among Nitrogen Oxides Reduction and N_2_ O Accumulation in Denitrification.” Environmental Science & Technology 47, no. 19: 11083–11091. 10.1021/es402348n. 24001217

[wer70439-bib-0015] Pang, Y. , and J. Wang . 2021. “Various Electron Donors for Biological Nitrate Removal: A Review.” Science of the Total Environment 794: 148699. 10.1016/j.scitotenv.2021.148699. 34214813

[wer70439-bib-0016] Peng, Y. , Y. Ma , and S. Wang . 2007. “Denitrification Potential Enhancement by Addition of External Carbon Sources in a Pre‐Denitrification Process.” Journal of Environmental Sciences 19, no. 3: 284–289. 10.1016/S1001-0742(07)60046-1. 17918588

[wer70439-bib-0017] Sun, H. , Q. Yang , Y. Peng , X. Shi , S. Wang , and S. Zhang . 2009. “Nitrite Accumulation During the Denitrification Process in SBR for the Treatment of Pre‐Treated Landfill Leachate.” Chinese Journal of Chemical Engineering 17, no. 6: 1027–1031. 10.1016/S1004-9541(08)60312-2.

[wer70439-bib-0018] Tchobanoglous, G. , ed. 2014. Wastewater Engineering: Treatment and Resource Recovery. Fifth ed. McGraw‐Hill Education.

[wer70439-bib-0019] United States Environmental Protection Agency (EPA) . 2009. National Primary Drinking Water Regulations. EPA 816‐F‐09‐004. [Online]. https://www.epa.gov/ground‐water‐and‐drinking‐water/national‐primary‐drinking‐water‐regulations.

[wer70439-bib-0020] Wang, J. , and L. Chu . 2016. “Biological Nitrate Removal From Water and Wastewater by Solid‐Phase Denitrification Process.” Biotechnology Advances 34, no. 6: 1103–1112. 10.1016/j.biotechadv.2016.07.001. 27396522

[wer70439-bib-0021] Wang, J. , X. Liu , A. H. W. Beusen , and J. J. Middelburg . 2023. “Surface‐Water Nitrate Exposure to World Populations Has Expanded and Intensified During 1970–2010.” Environmental Science & Technology 57, no. 48: 19395–19406. 10.1021/acs.est.3c06150. 38050814 PMC10702521

[wer70439-bib-0022] Ward, M. , R. Jones , J. Brender , et al. 2018. “Drinking Water Nitrate and Human Health: An Updated Review.” IJERPH 15, no. 7: 1557. 10.3390/ijerph15071557. 30041450 PMC6068531

[wer70439-bib-0023] Wu, W. , L. Yang , and J. Wang . 2013. “Denitrification Performance and Microbial Diversity in a Packed‐Bed Bioreactor Using PCL as Carbon Source and Biofilm Carrier.” Applied Microbiology and Biotechnology 97, no. 6: 2725–2733. 10.1007/s00253-012-4110-4. 22555916

[wer70439-bib-0024] Yang, X. , S. Wang , and L. Zhou . 2012. “Effect of Carbon Source, C/N Ratio, Nitrate and Dissolved Oxygen Concentration on Nitrite and Ammonium Production From Denitrification Process by *Pseudomonas stutzeri* D6.” Bioresource Technology 104: 65–72. 10.1016/j.biortech.2011.10.026. 22074905

[wer70439-bib-0025] Zhang, Y. , X. C. Wang , Z. Cheng , Y. Li , and J. Tang . 2016. “Effect of Fermentation Liquid From Food Waste as a Carbon Source for Enhancing Denitrification in Wastewater Treatment.” Chemosphere 144: 689–696. 10.1016/j.chemosphere.2015.09.036. 26408975

[wer70439-bib-0026] Zhu, Y. , Y. G. Zhao , J. Liu , et al. 2024. “Rapid Conversion of Heterotrophic Denitrification to Autotrophic Denitrification in Mariculture Wastewater Treatment: Denitrification Performance and Microbial Communities Under Antibiotic Stress.” Journal of Water Process Engineering 62: 105391. 10.1016/j.jwpe.2024.105391.

