# Supplementary material for: Decoupling Dissolution and Biological Kinetics in Polycaprolactone‐Based Denitrification: Direct Determination of Yield and Maximum Specific Growth Rate
Source: Water Environ Res. 2026 Jun 10;98(6):e70439. doi: 10.1002/wer.70439 (PMC13253049; doi:10.1002/wer.70439)
Supplement: Supplementary file 1 — Table S1: Stoichiometric COD/N ratios and electron requirements for sequential denitrification steps. [file WER-98-e70439-s001.docx]

**Table S1.** Stoichiometric COD/N ratios and electron requirements for sequential denitrification steps

| Denitrification Step | e^−^ needed per mole N | COD equivalent  (g O_2_) | COD/N ratio  (g COD/ g N) |
| --- | --- | --- | --- |
| NO_3_^−^ → NO_2_^−^ | 2 | 2 e^−^ × 8 = 16 | 16 / 14 = 1.14 |
| NO_2_^−^ → N_2_ | 3 | 3 e^−^ × 8 = 24 | 24 / 14 = 1.72 |
| NO_3_^−^ → N_2_ | 5 | 5 e^−^ × 8 = 40 | 40 / 14 = 2.86 |
